# Integrated model of Student Standardized Patients and peer assessment enhances SOAP note proficiency in obstetrics and gynecology residency

**DOI:** 10.3389/fmed.2026.1797822

**Published:** 2026-05-18

**Authors:** Peiqiong Chen, Yizhou Huang, Xiangrong Xu, Xiao Wang, Luyao Wu, Jianhong Zhou, Jingyi Li

**Affiliations:** 1 Department of Gynecology, Women’s Hospital, Zhejiang University School of Medicine, Hangzhou, China; 2 Department of Education, Women’s Hospital, Zhejiang University School of Medicine, Hangzhou, China

**Keywords:** education evaluation, peer assessment, standardized residency training, Student Standardized Patients, subjective-objective-assessment-plan

## Abstract

**Objective:**

To assess the efficacy of an integrated training model that incorporates Students as Standardized Patients (SSPs) alongside structured peer assessment in enhancing SOAP (subjective-objective-assessment-plan) note-writing proficiency and overall course satisfaction among obstetrics and gynecology residents.

**Design:**

A prospective controlled educational study was conducted from January 2024 to June 2025. Third-year residents were allocated to either an experimental group (*n* = 51), which received training incorporating SSPs combined with peer assessment within a small-group collaborative setting, or a control group (*n* = 54), which received traditional peer role-play training. The outcomes were evaluated using the self-developed Teaching Experience and Satisfaction Questionnaire (TESQ), assessing seven domains on a 5-point Likert scale, and standardized SOAP note scores derived from the official Zhejiang Province Standardized Residency Training Clinical Practice Competency Rubric.

**Results:**

The experimental group demonstrated significantly greater satisfaction across six of the seven evaluated domains, with the most pronounced differences observed in Classroom Organization, Clinical Reasoning Development, and Model Recommendation (all *P* < 0.01). Furthermore, the total SOAP note scores were significantly higher in the experimental group (88.25 ± 2.76) than in the control group (85.20 ± 3.01; *P* < 0.01). Subsection analysis indicated that these improvements were primarily attributable to enhanced performance in the Objective (O) and Assessment (A) sections, likely reflecting the model’s effectiveness in strengthening structured clinical data acquisition and analytical reasoning through interactive simulation and feedback. In contrast, no significant differences were detected in the Subjective (S) and Plan (P) sections. The final assessment pass rates did not differ significantly between the groups.

**Conclusion:**

The incorporation of an integrated SSP and peer assessment model improved both the subjective training experience and objective performance in SOAP note writing within residency education. These improvements were particularly evident in domains requiring structured clinical data interpretation and reasoning, suggesting that the model preferentially enhances higher-order cognitive skills. This model constitutes a viable and resource-efficient innovation that bolsters clinical data collection and analytical reasoning. Furthermore, it provides a replicable approach for enhancing competency-based training in resource-constrained settings in the future.

## Introduction

Residency training constitutes a pivotal stage in postgraduate medical education, designed to develop proficient physicians equipped for autonomous clinical practice. A critical competency developed during this training is the ability to compose an initial clinical note utilizing the subjective-objective-assessment-plan (SOAP) format. Originally introduced by Dr. Lawrence Weed in the 1960s (1), this internationally recognized standardized system for documenting patient symptoms, clinical findings, diagnoses, and treatment plans has become pervasive in clinical practice (2). The importance of this component is further emphasized by its inclusion in the final competency assessments for residency training, as mandated by national guidelines, such as the Implementation Measures for Residency Training Assessment by China’s National Health Commission, and regional requirements, such as the Clinical Practice Skill Assessment Standards of Zhejiang Province.

Despite its significance, many training hospitals frequently rely on conventional pedagogical methods for instructing SOAP note writing (3). These methods generally involve trainees practicing with peer-simulated patients within their groups. However, owing to the limited knowledge base and clinical experience of simulated peers, the efficacy of such training is often suboptimal. Consequently, there is an increasing trend toward adopting more interactive, inquiry-based, and experiential teaching models (4, 5). These models aim to foster proactive learning, harness student potential, and enhance their abilities for independent critical thinking and practical problem-solving–skills that are essential in clinical practice.

The situational teaching methodology addresses the need for effective learning by utilizing authentic or simulated scenarios pertinent to educational content, thereby promoting active knowledge construction and enhancing practical skills through interactions (6). A prevalent application of this methodology is the use of Standardized Patients (SPs). However, the implementation of SPs is often hindered by substantial demands on financial resources, faculty involvement, and the extensive time required for SP recruitment and training (7). In many instances, the high costs associated with SPs can limit their widespread adoption, especially in resource-constrained settings. An alternative approach involves the use of Students as Standardized Patients (SSPs), which offers several advantages. SSPs are characterized by their ability to rapidly acquire knowledge and require significantly less training time, thereby providing medical trainees with more frequent opportunities to practice their clinical skills (8). Moreover, participation as an SSP enriches students’ comprehension of disease presentations and management strategies while simultaneously enhancing their communication skills with patients (9). This approach has been shown to improve students’ clinical and interpersonal skills without the logistical challenges posed by SP programs.

In conjunction with situational teaching, peer assessment (PA) is a pedagogical process whereby students, serving as evaluators, assess the work of their peers based on established criteria and offer constructive feedback. Extensive research substantiates that PA promotes reciprocal learning, encourages reflective and critical thinking, and ultimately improves overall student competency (10, 11).

Given these challenges and the recognized benefits of both SSPs and PA, this study aimed to integrate the SSP method with structured PA into the SOAP note-writing curriculum. Our objective was to evaluate the effectiveness of this combined approach in enhancing resident competencies within a resource-efficient framework, addressing recurrent resource limitations in clinical education and the critical need to cultivate self-regulation, teamwork, systems thinking, and ethical judgment.

We hypothesized that, compared with traditional training, the integrated SSP and peer assessment model would significantly improve residents’ SOAP note-writing performance, particularly in domains requiring objective data interpretation and clinical reasoning, while also enhancing learner satisfaction within a resource-efficient educational framework.

## Materials and methods

### Participants

This study involved third-year obstetrics and gynecology residents who commenced their training in 2021 and 2022 at the Women’s Hospital, Zhejiang University School of Medicine. The study was conducted from January 2024 to June 2025. A total of 105 residents participated in the study, forming the study cohort. This cohort was stratified into two groups according to the year of enrollment. Residents who enrolled in 2021 (*n* = 54; 5 males, 49 females) were designated as the control group, whereas those who enrolled in 2022 (*n* = 51; 5 males, 46 females) comprised the experimental group. The training intervention and outcome assessments for these cohorts were conducted in 2024 and 2025, respectively. Throughout their clinical rotations, residents from both groups were organized into small training groups of 8–9 individuals within the same departments. Each participant in a group was assigned a sequential identifier.

Given that group allocation was based on enrollment year rather than randomization, potential cohort-related differences may exist between groups.

### Study design

Control group: traditional peer role-play training. Residents in the control group underwent training using a conventional pedagogical approach. The session began with the instructor delivering a succinct overview of the theoretical foundations and essential elements of SOAP note writing. This was followed by a demonstration interview in which the instructor designated one resident to assume the role of the patient. Following this demonstration, residents within each small group engaged in practice sessions, wherein they conducted interviews and composed notes, with their peers taking turns to act as simulated patients. At the conclusion of the practice session, the resident who assumed the role of the patient during a specific practice exchange evaluated the SOAP note authored by their peer, who acted as the interviewer. This evaluation was performed using the official Zhejiang Province Standardized Residency Training Clinical Practice Competency Final Assessment SOAP Score Sheet (12).

Experimental group: SSPs with PA Model. Following a uniform theoretical review, residents in the experimental group engaged in interviews within their small cohorts alongside trained Students as Standardized Patients (SSPs) who rigorously adhered to their scripted roles. Post-interview, each resident was tasked with composing a SOAP Score Sheet. Thereafter, all members of the group participated in a structured peer assessment session, utilizing the standardized Scoring Standards to evaluate the SOAP scores of their peers.

Students as Standardized Patients Preparation and Training: six second-year residents were recruited and underwent formal training to serve as Standardized Simulated Patients (SSPs). Experienced clinical faculty from the teaching department selected six representative case scripts from the hospital’s obstetrics and gynecology standardized patient library. Trainers provided guidance to the SSP trainees on script interpretation, role embodiment, and specific response protocols. Comprehensive explanations of all the performance metrics and assessment criteria are provided. SSPs were authorized to commence their duties only after successfully passing a qualification assessment.

### Questionnaire

After the training, participants completed a structured, self-developed questionnaire–the Teaching Experience and Satisfaction Questionnaire (TESQ)–administered independently of final assessments to minimize social desirability and evaluation apprehension. The TESQ assessed seven domains: instructors’ organizational competence, course ability to stimulate learning interest, facilitation of teamwork, enhancement of physician–patient communication skills, effectiveness in improving knowledge application, contribution to clinical reasoning development, and participants’ willingness to recommend the teaching model for broader implementation. Items were rated on a 5-point Likert scale (1 = strongly disagree, 5 = strongly agree).

The TESQ was developed through a systematic process, including a review of relevant literature and expert consultation. Content validity was ensured via review by a panel of three senior medical educators, who evaluated each item for relevance and clarity, with low-rated items revised or removed based on feedback. Internal consistency reliability of the TESQ was assessed using Cronbach’s alpha, yielding 0.85, indicating high reliability. Although these instruments demonstrated content validity and reliability within this study, they have not undergone comprehensive external psychometric testing, which represents a limitation.

#### Final assessment for residents

**FIGURE 1:**
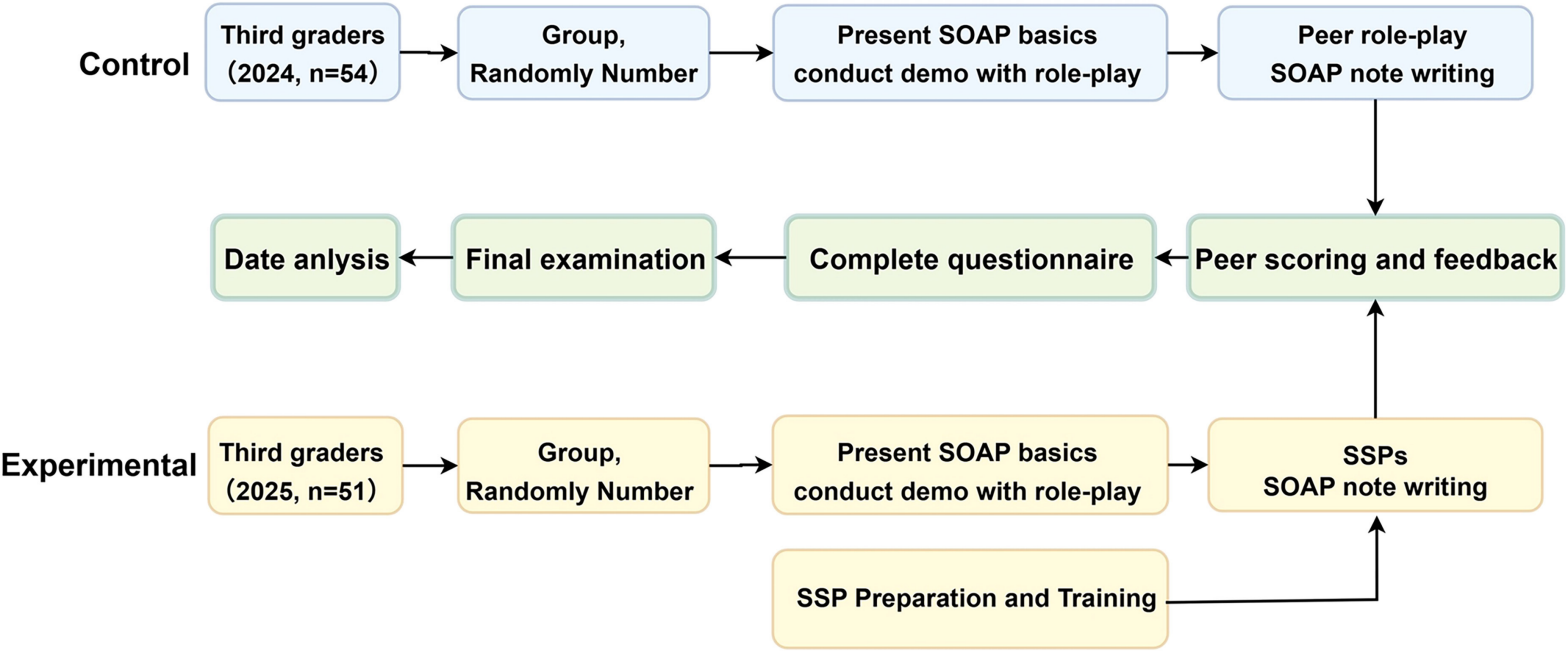
Flow diagram of the study. The control and experimental groups correspond to residents enrolled in 2021 and 2022, respectively. The years 2024 and 2025 shown in the figure represent the time points at which the training intervention and assessments were conducted.

All the third-year residents would ultimately take the Zhejiang Provincial Obstetrics and Gynecology Residency Standardized Training Final Examination, which consists of an Objective Structured Clinical Examination (OSCE) for clinical skills and a theoretical examination. The study flow diagram is depicted in Figure 1.

#### Data analysis

Statistical analyses were performed using SPSS software (version 22.0). Continuous variables are presented as mean ± standard deviation (SD), and categorical variables are expressed as frequencies and percentages. Comparisons between groups were conducted using independent-samples *t*-tests for continuous variables and chi-square (χ^2^) tests for categorical variables. A two-tailed *P* < 0.05 was considered statistically significant. Graphs were generated using GraphPad Prism version 8.0.2.

#### Ethical issues

The protocol was conducted in accordance with the Declaration of Helsinki and received approval from the Human Ethics Committee of Women’s Hospital, Zhejiang University School of Medicine (No. IRB-20260077-R). Informed consent was obtained from all participants.

## Results

The study comprised 54 participants in the control group (5 men and 49 women) and 51 participants in the experimental group (5 men and 46 women), thereby affirming the comparability of the groups at the baseline (Table 1). Having established group comparability, we proceeded to assess the primary outcomes of the integrated training intervention.

**TABLE 1:** Student characteristics.

Item	Category	Control group	Experimental group
Gender	Male	5 (9.25%)	5 (9.80%)
	Female	49 (90.75%)	46 (90.20%)
Grade	Third-year	100%	100%
Age	25–30 years	45 (83.33%)	41 (80.39%)
	≥30 years	9 (16.67%)	10 (19.61%)
Origin	Employers	35 (64.82%)	34 (66.67%)
	Master’s student	17 (31.58%)	15 (29.41%)
	Social personnel	2 (3.70%)	2 (3.92%)

Teaching Experience and Satisfaction Questionnaire scores were evaluated across seven domains (Table 2). Group comparisons were performed using independent-samples *t*-tests, and results are presented as mean ± SD. Satisfaction scores for Classroom Organization were significantly higher in the experimental group (4.80 ± 0.40) than in the control group (4.56 ± 0.50; *t* = 2.79, *P* = 0.006). Similarly, marked differences (*P* < 0.01) were observed for Clinical Reasoning Development (experimental: 4.65 ± 0.52 vs. control: 4.37 ± 0.48; *t* = 2.807) and Model Recommendation (experimental: 4.25 ± 0.65 vs. control: 3.85 ± 0.65; *t* = 3.140). The experimental group also reported significantly higher satisfaction (*P* < 0.05) in three further domains: Stimulation of Learning Interest (4.55 ± 0.50 vs. 4.31 ± 0.50; *t* = 2.375, *P* = 0.020), physician–Patient Communication (4.55 ± 0.50 vs. 4.32 ± 0.53; *t* = 2.291, *P* = 0.024), and Knowledge Application (4.51 ± 0.54 vs. 4.33 ± 0.51; *t* = 2.081, *P* = 0.040). In contrast, no significant difference was found between the groups for Teamwork Cultivation (experimental: 4.49 ± 0.54 vs. control: 4.37 ± 0.55; *t* = 1.113, *P* = 0.269), indicating that the intervention did not substantially affect perceived satisfaction in this area.

**TABLE 2:** Results of the Teaching Experience and Satisfaction Questionnaire.

Question item	Control group	Experimental group	t	*P*
Classroom organization	4.56 ± 0.50	4.80 ± 0.40**	2.79	0.006
Stimulation of learning interest	4.31 ± 0.50	4.55 ± 0.50*	2.375	0.020
Teamwork cultivation	4.37 ± 0.55	4.49 ± 0.54	1.113	0.269
Physician-patient communication	4.32 ± 0.53	4.55 ± 0.50*	2.291	0.024
Knowledge application	4.33 ± 0.51	4.51 ± 0.54*	2.081	0.040
Clinical reasoning development	4.37 ± 0.48	4.65 ± 0.52**	2.807	0.006
Model recommendation	3.85 ± 0.65	4.25 ± 0.65**	3.140	0.002

Data are presented as mean ± SD. *P < 0.05, **P < 0.01.

**FIGURE 2:**
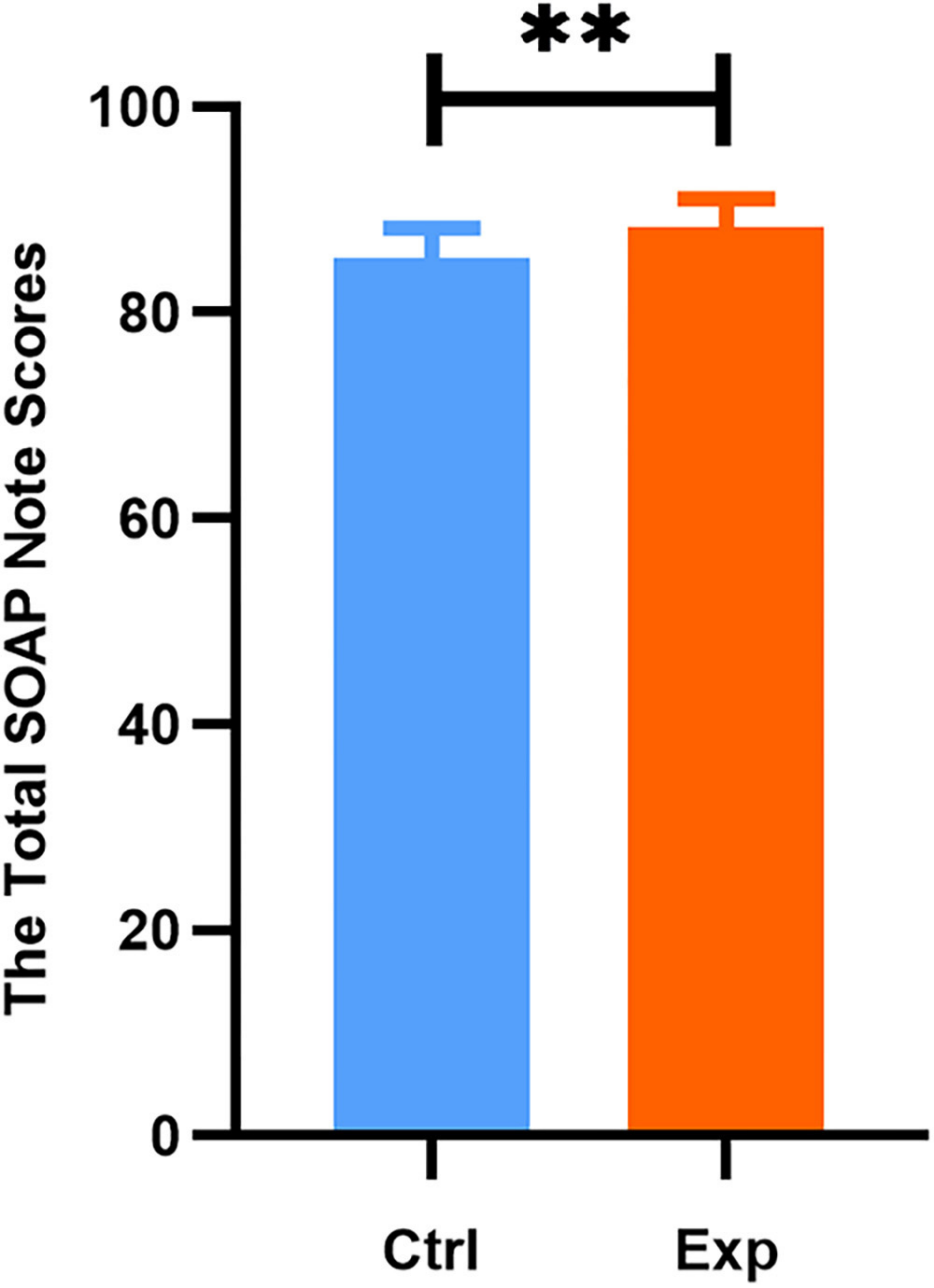
Overall performance in SOAP across the two groups. Ctrl, control group; Exp, experimental group. Data are presented as mean ± SD. **P < 0.01.

**FIGURE 3:**
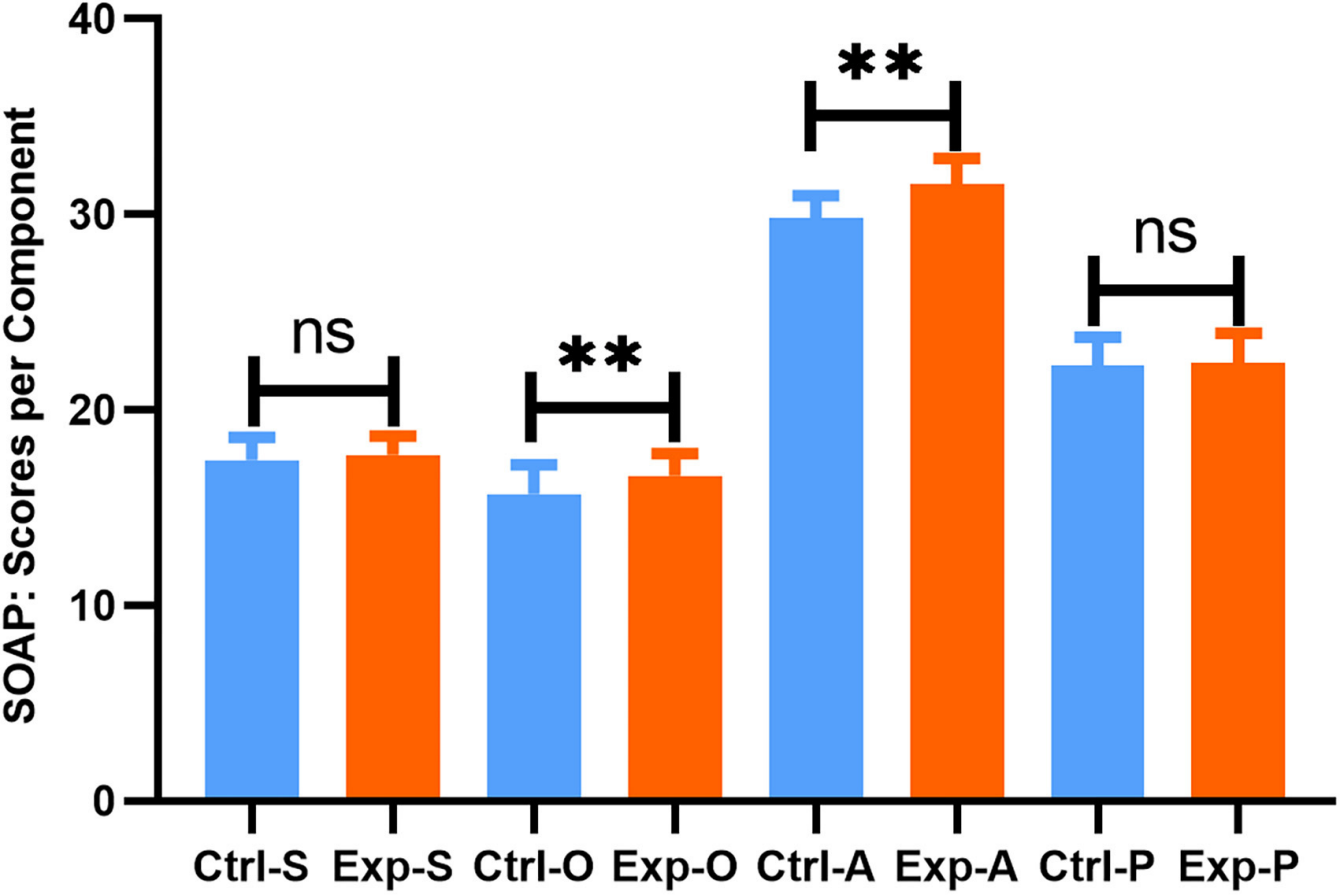
Analysis of SOAP component scores by group. Ctrl, control group; Exp, experimental group. Data are presented as mean ± SD. **P < 0.01.

Beyond trainee satisfaction, we also assessed the objective performance in SOAP note writing. Participants from both cohorts assessed the SOAP Scores authored by their consecutively numbered peers using the SOAP Score Sheet. The evaluation rubric allocated weights to each section as follows: subjective (S), 20%; objective (O), 20%; assessment (A), 35%; and plan (P), 25%. The overall SOAP score was markedly higher in the experimental group (88.25 ± 2.76) than in the control group (85.20 ± 3.01; *P* < 0.01; Figure 2). Although no significant differences were observed between the two groups in the Subjective (S) and Plan (P) sections, the experimental group attained significantly higher scores in the Objective (O) (experimental: 16.63 ± 1.1 vs. control: 15.69 ± 1.49; *t* = 3.668, *P* = 0.0004) and Assessment (A) (experimental: 31.53 ± 1.32 vs. control: 29.81 ± 1.12; *t* = 7.182, *P* < 0.0001) sections relative to the control group (Figure 3).

**FIGURE 4:**
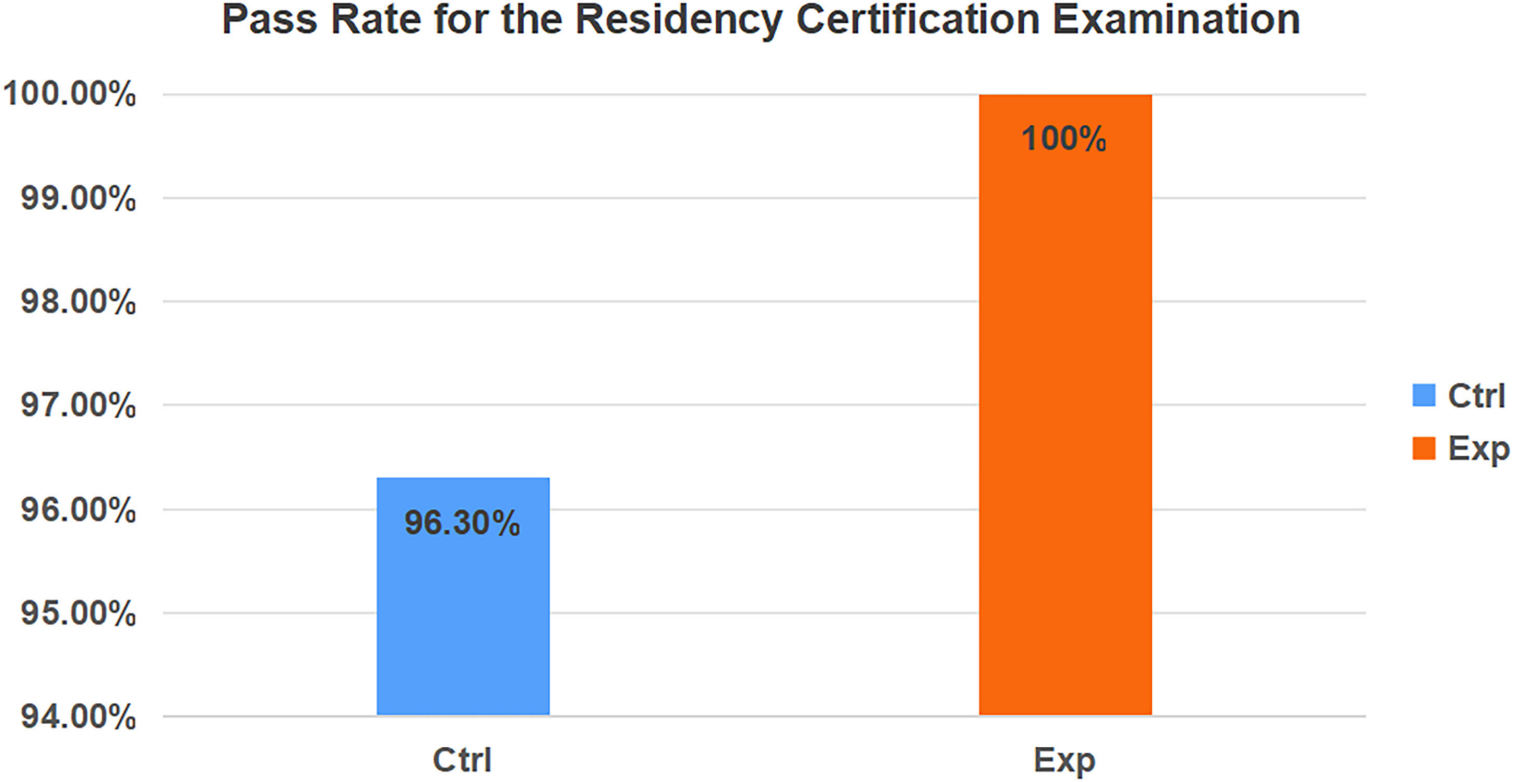
Comparison of certification exam pass rates among resident cohorts. Ctrl, control group; Exp, experimental group.

The final competency assessment pass rates were 52 out of 54 (96.30%) for the control group and 51 out of 51 (100%) for the experimental group, respectively. Nonetheless, the analysis did not reveal a statistically significant difference in pass rates between the two groups (Figure 4).

## Discussion

This prospective, controlled teaching study assessed the impact of an integrated educational model, which combined the use of Students as Standardized Patients (SSPs) with structured peer assessment within a small-group collaborative learning framework, on the efficacy of SOAP note-writing training for obstetrics and gynecology residents. The findings indicate that, compared with the traditional small-group collaborative approach, the integrated model significantly enhanced both trainees’ satisfaction with the course and their performance on the SOAP note assessment. Notably, the most substantial improvements were observed in core competency areas, such as the collection of objective data and clinical assessments. These findings suggest that the model may be particularly effective in strengthening structured clinical reasoning and data acquisition skills.

This study supports the effectiveness of the integrated “SSP + peer assessment” model in developing SOAP note-writing competency. The combination of standardized clinical scenarios and structured peer feedback created a synergistic learning environment, enhancing both engagement and higher-order cognitive processes. Consistent with previous studies, the experimental group demonstrated higher satisfaction across domains such as learning interest, communication skills, knowledge application, and clinical thinking. These findings suggest that the model not only improves procedural skills but also promotes deeper learning through increased motivation and active participation.

The differential effects observed across SOAP components provide further insight into the educational mechanisms of the intervention. The experimental group showed significant improvement in the Objective (O) and Assessment (A) sections, suggesting enhanced performance in structured data collection and clinical reasoning. Traditional peer role-play, often limited by non-standardized case portrayal and variable clinical experience, may be less effective in training these competencies (13). In contrast, SSPs offer consistent and reproducible scenarios that better support the development of examination skills and diagnostic reasoning. Furthermore, the structured peer-assessment component necessitated that the residents critically engage with the official scoring rubric (14). This process likely facilitated the internalization of evaluation standards, thereby improving both the standardization of documentation and the rigor of clinical reasoning. No significant differences were observed in the Subjective (S) and Plan (P) sections. This may reflect the relatively high baseline proficiency in history-taking and the greater dependence of planning skills on domain-specific knowledge and clinical experience, which may be less responsive to short-term educational interventions (15, 16).

Beyond learning outcomes, this study highlights a practical and resource-efficient educational approach. By training senior residents as SSPs, the model reduces the financial and logistical burden associated with professional Standardized Patients (17). Moreover, the SSP training methodology itself acts as an effective tool for enhancing participants’ clinical acumen, communication abilities, and empathy, thus exemplifying the educational principle of “mutually reinforcing teaching and learning” (18).

In addition, the integration of peer assessment transforms evaluation from a solely summative process into a more formative and learner-centered approach. This not only reduces faculty workload but also encourages residents to critically appraise clinical documentation and engage in reflective learning. Such processes have been shown to enhance understanding of quality standards and promote self-regulated learning (19).

Despite these strengths, several limitations and potential sources of bias should be considered when interpreting the findings. First, being a single-center study with a limited sample size concentrated exclusively on obstetrics and gynecology, the generalizability of the results to other medical specialties or different hospital settings requires further validation. Second, the assessment of teaching effectiveness was predominantly based on immediate post-course satisfaction surveys and rubric-based writing scores, which do not provide direct evidence of long-term skill retention or behavioral changes in actual clinical practice. Third, as group allocation was based on enrollment cohorts rather than randomization, potential cohort effects could not be fully eliminated, despite efforts to standardize teaching conditions. In addition, although peer assessment was guided by a standardized rubric, subjective judgment and interpersonal dynamics may have introduced bias. The absence of independent external raters for SOAP note evaluation may have further limited the objectivity of performance assessment. Fourth, the SOAP scoring rubric, while developed by an expert panel and widely used in regional high-stakes examinations, has not undergone formal psychometric validation. Similarly, the questionnaire, while content-validated, has not undergone comprehensive psychometric testing. This underscores the necessity for more robust quality control mechanisms in future implementations.

This study identifies several promising avenues for future research. First, the refinement of the SSP training system and case script library is recommended, alongside the expansion of SSP applications to encompass more complex scenarios, such as challenging communication encounters and ethical dilemmas. Second, future research could investigate the integration of digital tools, including recorded interview videos and online interactive peer review platforms, to enhance the objectivity, traceability, and quality of feedback in peer assessments. Finally, conducting longitudinal follow-up studies to evaluate the impact of this integrated model on ultimate outcomes, such as post-training clinical performance and patient-related results, would provide more robust evidence of its educational value.

## Conclusion

In conclusion, this study suggests that the integration of Students as Standardized Patients, combined with structured peer assessment within a small-group collaborative learning framework, can enhance both SOAP note writing proficiency and training satisfaction among obstetrics and gynecology residents. This model represents a feasible and resource-efficient approach to teaching essential clinical skills and may serve as a useful reference for optimizing pedagogical strategies in standardized residency training programs. However, further studies with more rigorous designs and broader settings are warranted to confirm these findings.

## Data Availability

The original contributions presented in this study are included in this article/supplementary material, further inquiries can be directed to the corresponding authors.
